# Structure-guided design of a high-affinity ligand for a riboswitch

**DOI:** 10.1261/rna.069567.118

**Published:** 2019-04

**Authors:** Lin Huang, Jia Wang, Timothy J. Wilson, David M.J. Lilley

**Affiliations:** Cancer Research UK Nucleic Acid Structure Research Group, MSI/WTB Complex, The University of Dundee, Dundee DD1 5EH, United Kingdom

**Keywords:** RNA ligand design, molecular recognition, riboregulation, X-ray crystallography

## Abstract

We have designed structure-based ligands for the guanidine-II riboswitch that bind with enhanced affinity, exploiting the twin binding sites created by loop–loop interaction. We synthesized diguanidine species, comprising two guanidino groups covalently connected by C_n_ linkers where *n* = 4 or 5. Calorimetric and fluorescent analysis shows that these ligands bind with a 10-fold higher affinity to the riboswitch compared to guanidine. We determined X-ray crystal structures of the riboswitch bound to the new ligands, showing that the guanidino groups are bound to both nucleobases and backbone within the binding pockets, analogously to guanidine binding. The connecting chain passes through side openings in the binding pocket and traverses the minor groove of the RNA. The combination of the riboswitch loop–loop interaction and our novel ligands has potential applications in chemical biology.

## INTRODUCTION

RNA provides a versatile scaffold for binding small molecule ligands with high selectivity. This is particularly well illustrated by the riboswitches (Roth and Breaker 2009; Serganov and Nudler 2013), *cis*-acting regulatory elements that occur in the 5′ noncoding regions of (mostly) bacterial mRNA that are widely used to control gene expression. Many classes have now been identified that respond to a range of metabolites including coenzymes, amino acids, purines, and even ions. Atomic resolution structures are available for many riboswitches, and this provides an opportunity to carry out ligand engineering to design new species that will bind with elevated affinity. In this work we have used a structure-guided approach that uses a unique feature of a guanidine riboswitch to create a novel ligand.

The *ykkC* riboswitches comprise a group of three structurally unrelated riboswitches that bind guanidine. Breaker and colleagues (Nelson et al. 2017) showed that ligand binding to these riboswitches up-regulates the expression of a series of genes whose products either chemically convert guanidine or export it from the cell. Three *ykkC* types have been identified, called the guanidine-I (Nelson et al. 2017), -II (Sherlock et al. 2017), and -III (Sherlock and Breaker 2017) riboswitches. Crystal structures have been solved for members of each class (Battaglia et al. 2017; Huang et al. 2017a,b; Reiss and Strobel 2017; Reiss et al. 2017). We have exploited a novel feature of the guanidine-II riboswitch structure to design a high-affinity ligand.

The guanidine-II riboswitch comprises two stem–loops with G+C-rich helices and an ACGR (R = A or G) tetraloop, connected by a short polynucleotide of ∼14 nt (Sherlock et al. 2017). Individual single stem–loops undergo loop–loop interaction driven by the cooperative binding of guanidine. Using X-ray crystallography, our laboratory (Huang et al. 2017a) and that of Strobel (Reiss and Strobel 2017) showed that the stem–loops dimerize by loop–loop interaction involving the formation of intermolecular base pairs and triples. Formation of the dimer creates identical binding sites for two guanidine ligands symmetrically. These are bound by donation of guanidine protons to O6 and N7 of a guanine nucleobase (G9 in our usual numbering scheme [Huang et al. 2017a]), and to nonbridging oxygen atoms of consecutive phosphate groups. At neutral pH guanidine is protonated, thus having six protons and *D*_3h_ symmetry. Four of these protons are involved in specific interactions with the RNA. Protonation confers a positive charge (i.e., it is more properly called the guanidinium cation), and the ligand is stacked upon the guanine nucleobase of the loop-proximal base pair (G6–C11), so that the cation–π interaction contributes to the stability of the dimer.

In this report, we demonstrate how a structure-guided rational approach can be used to re-engineer the ligand of the guanidine-II riboswitch, exploiting its unusual creation of two binding sites by loop–loop interaction. Sherlock et al. (2017) found that some variants of guanidine with substitution on one nitrogen atom such as methylguanidine and aminoguanidine bound to the guanidine-II riboswitch with affinities that were within a factor of four of guanidine itself. Furthermore, even addition of a butylamine side chain (agmatine) led to retention of binding despite a further loss of affinity. Our crystal structure of the *Gloeobacter violaceus* guanidine-II riboswitch (Huang et al. 2017a) revealed that the binding pocket had a side opening that might accommodate one or more additional atoms attached to one nitrogen. We diffused these compounds into our crystals, and obtained structures of the riboswitch bound to the modified guanidine species (Huang et al. 2017a). This revealed that the guanidino moiety was bound in exactly the same way as guanidine, and that the additional methyl, amino and butylamine side chains did indeed emerge from the side pocket. In the latter case while the electron density for the guanidine was clear, that for the longer side chain was poorly defined suggesting that it became progressively more mobile as it emerged from the side opening.

The side openings of the two guanidine binding sites in the stem–loop dimer are ∼7 Å apart and oriented toward each other on the minor groove side of the loop–loop interface, so we wondered if two guanidino moieties might be covalently linked to create a higher-affinity ligand. Molecular modeling based on our structure of guanidine-bound riboswitch suggested that two guanidine units linked by C_4_ or C_5_ polymethylene chains should bind to the riboswitch.

We therefore set out to synthesize C_4_- and C_5_-linked diguanidine species and examine their binding to the *G. violaceus* guanidine-II riboswitch. We have found that both compounds bind to the riboswitch with enhanced affinity and lower stoichiometry. We crystallized the two bound complexes and solved their structures, showing that the linked ligands bind in the anticipated manner.

## RESULTS AND DISCUSSION

### Synthesis of diguanidine species

**FIGURE 1.**
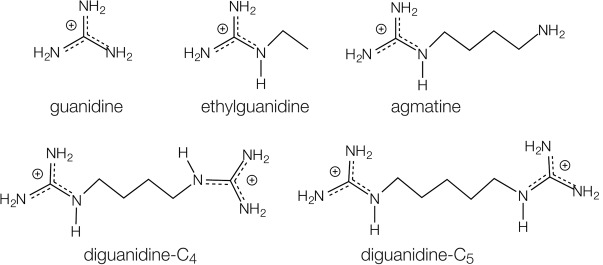
Chemical structures of guanidine, modified guanidine species, and diguanidine species discussed in this work. The parental guanidine is shown *top left*, followed by ethylguanidine and agmatine that are modified by addition of ethyl and butylamine groups, respectively. *Below* are shown the diguanidine-C_4_ and diguanidine-C_5_ species in which two guanidine groups are connected by C_4_ and C_5_ chains, respectively. In all cases, the guanidino moieties are shown as guanidinium cations that are the abundant form at neutral pH.

In order to explore the binding properties of linked guanidines, we synthesized two forms of diguanidine species in which the guanidine moieties are covalently connected by four or five carbon atoms (Fig. 1). For clarity, we term these species diguanidine-C_4_ [N,N′-(butane-1,4-diyl)bis guanidinium, also known as arcaine] and diguanidine-C_5_ [N,N′-(pentane-1,5-diyl)bis guanidinium also known as audouine] respectively. The synthetic procedures are detailed in Materials and Methods. In brief, diguanidine-C_4_ and diguanidine-C_5_ were synthesized by guanylation of (4-aminobutyl)guanidine (agmatine) and 1,5-diaminopentane (cadaverine) using 1 and 2 molar equivalents of 1-H-pyrazole-1-carboxamidine hydrochloride (Bernatowicz et al. 1992), respectively (Supplemental Fig. S1A). The products were characterized by ^1^H NMR and mass spectrometry (Supplemental Fig. S1B,C), although the crystal structures of the complexes (see below) provide unambiguous evidence that these compounds have the required structure.

### Calorimetric analysis of binding of guanidine and diguanidine species to the guanidine-II riboswitch

**FIGURE 2.**
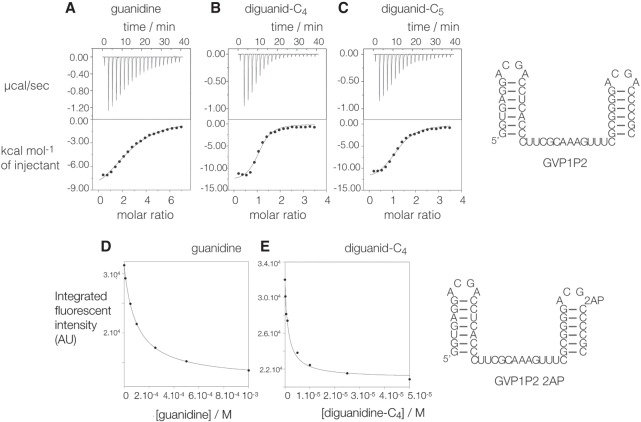
Binding of guanidine and diguanidine species to the complete *G. violaceous* guanidine-II riboswitch studied by isothermal titration calorimetry and fluorescence spectroscopy. (*A–C*) Calorimetry. A solution of ligand was titrated into the RNA solution, and the heat evolved was measured as the power required to maintain zero temperature difference with a reference cell. Integration over time gives the heat required to maintain thermal equilibrium between cells. In each case, the *upper* panel shows the raw data for sequential injections of 2 µL volumes (following an initial injection of 0.4 µL) of ligand into 200 µL of a 15 µM RNA solution in 40 mM HEPES (pH 7.2), 100 mM KCl, 10 mM MgCl_2_. This represents the differential of the total heat (i.e., enthalpy Δ*H*° under conditions of constant pressure) for each ligand concentration. The *lower* panels present the integrated heat data fitted to a one-set-of-sites binding model. The thermodynamic parameters calculated are summarized in Supplemental Table S1. Binding was studied using the ligands guanidine (*A*), diguanidine-C_4_ (*B*), and diguanidine-C_5_ (*C*). The sequence of the riboswitch is shown on the *right*. Titration of individual P1 and P2 stem–loops is shown in Supplemental Figure S2. (*D*,*E*) Ligand-induced folding of the guanidine riboswitch studied by 2-aminopurine fluorescence. The GVP1P2 construct used in these experiments (the sequence is shown on the *right*) contains a single A10 2-aminopurine (2AP) at the 3′ end of the loop. On binding, the ligand loop–loop interaction generates A10–A10′ stacking in the crystal, and results in static quenching of 2-aminopurine fluorescence. Fluorescence emission spectra (λ_excite_ = 315 nm; λ_emission_ = 340–450 nm) were recorded as a function of added ligand concentration using guanidine and diguanidine-C_4_. Fluorescence intensity was integrated between 355 and 375 nm, and plotted as a function of guanidine (*D*) and diguanidine-C_4_ (*E*) concentration. Ligand binding was also studied by ITC, shown in Supplemental Figure S2.

We have investigated the binding of guanidine, diguanidine-C_4_, and diguanidine-C_5_ to individual guanidine-II riboswitch stem–loops and a complete riboswitch using isothermal titration calorimetry (ITC) (Fig. 2; Supplemental Fig. S2). First, we have studied the individual P1 and P2 stem–loops derived from *G. violaceous*, for which we have previously determined crystal structures (Huang et al. 2017a). Titration curves are presented in Supplemental Figure S2. The binding of guanidine to the P1 and P2 stem–loops is an exothermic, enthalpy-dominated reaction. Fitting the titration curves gives dissociation constants (*K*_d_) for the binding reaction of 68 µM and 66 µM, respectively, at molar ratios *n* = 1.9 and 2.2 (Supplemental Table S1). A stoichiometry of *n* = 1 is expected for the binding of guanidine to the isolated stem–loops, but crystal structures reveal a second guanidine binding site near the top of the stem–loop. Reiss and Strobel (2017) observed spermidine and a hydrated magnesium ion bound to the same site, which evidently has a tendency to bind cationic ligands, including guanidine. The additional binding site may account for the observed molar ratio of *n* ∼ 2, in which case it must bind with a similar affinity. Calorimetric titration was repeated for diguanidine-C_4_, giving values of *K*_d_ = 4.7 and 5.9 µM for P1 and P2, respectively, each with a molar ratio of *n* = 0.4, close to the expected stoichiometry of 0.5. Thus, linking the guanidine ligands increases the affinity by an order of magnitude compared to guanidine.

We have further explored the binding of guanidine, diguanidine-C_4_, and diguanidine-C_5_ to a complete *G. violaceous* riboswitch with linked P1 and P2 stem loops using ITC (Fig. 2A–C). As with the individual stem–loops, all three compounds exhibit exothermic binding, with *K*_d_ = 33, 2.2, and 5.1 µM, respectively (Supplemental Table S1). Binding exhibited molar ratios of *n* = 2.7, 1.1 and 1.2, respectively. The binding affinities for each compound are approximately twofold higher for the complete riboswitch compared to the individual stem–loops. Furthermore, as with the individual stem–loop structures, the diguanidine species bind an order of magnitude more tightly than guanidine and diguanidine-C_4_ binds with the highest affinity. The results are consistent with the binding of multiple molecules of guanidine to the riboswitch and a single molecule of the diguanidine species with ∼10-fold higher affinity.

### Spectroscopic analysis of binding of diguanidine species to the complete guanidine-II riboswitch

A feature of the dimerization of the stem–loops observed in the crystal was mutual stacking of the A10 nucleobases, that is, the 3′-terminal nucleotide of the loop. We have exploited this interaction to construct a spectroscopic probe of the loop–loop interaction between the stem–loops of the complete *G. violaceous* riboswitch. We synthesized a riboswitch in which A10 of the P2 loop was replaced by 2-aminopurine. This nucleobase is fluorescent, but subject to marked static quenching when stacked with another nucleobase. We therefore anticipated that the 2-aminopurine fluorescence intensity might decrease upon binding guanidine or related ligands that induce loop–loop interaction.

The 2-aminopurine-substituted riboswitch was titrated separately with guanidine and diguanidine-C_4_, recording the emission spectrum of 2-aminopurine excited at 315 nm. Addition of the ligands led to reduced fluorescence. The fluorescence intensity was integrated between 355 and 375 nm, and plotted as a function of ligand concentration (Fig. 2D,E). Addition of guanidine leads to a threefold quenching of 2-aminopurine fluorescence, and fitting the observed intensities to a two-state binding model gives *K*_d_ = 53 µM (Fig. 2D). 2-aminopurine fluorescence was also quenched on addition of diguanidine-C_4_, and fitting the intensity data led to a calculated affinity of *K*_d_ = 1.4 µM (Fig. 2E). The data are consistent with an intramolecular loop–loop interaction in the riboswitch, involving stacking of the adenine and 2-aminopurine at the 10 positions leading to quenching of the latter.

We have studied the ligand-induced folding of the 2-aminopurine-containing riboswitch in response to the addition of guanidine and diguanidine-C_4_ by ITC (Supplemental Fig. S2F,G). The ligands bound with an affinity of *K*_d_ = 41 and 6.6 µM, with molar ratios of *n* = 4.3 and 0.79, respectively.

### A crystal structure of the guanidine-II riboswitch bound to ethylguanidine

**FIGURE 3.**
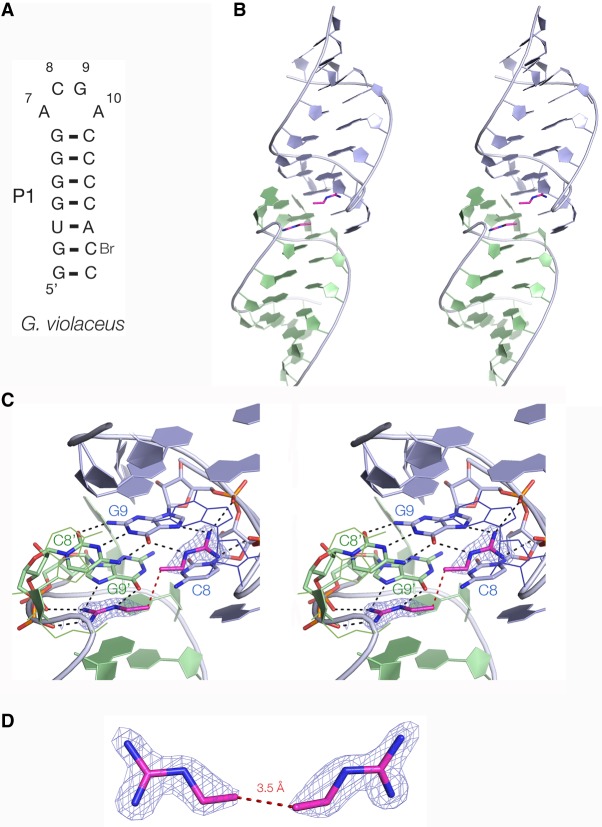
Crystal structure of ethylguanidine bound to *G. violaceous* riboswitch P1 stem–loop. (*A*) The sequence of the P1 stem–loop. The nucleotide numbering preserves the A7 to A10 numbering of the loop used previously (Huang et al. 2017b). (*B*) The overall structure shown in parallel-eye stereoscopic view. The P1 stem–loop forms a dimer by loop–loop interaction; the individual monomeric RNA species are colored here as blue and green. This color scheme is also used in Figure 4. The ethylguanidine molecules are colored magenta. (*C*) Parallel-eye stereoscopic view of the two bound ethylguanidine molecules bound at the dimer interface, with electron density (2*F*_o_−*F*_c_) contoured at 2σ shown for the ligands. The ethylguanidine molecules are hydrogen bonded to G9 and G9′ and nonbridging phosphate oxygens of the backbone. (*D*) The two ethylguanidine molecules with their experimental phasing electron density map contoured at 1σ. The two terminal carbon atoms are separated by 3.3 Å (broken red line); these would be connected by a single C-C bond in diguanidine-C_4_.

Ethylguanidine can be regarded as half of diguanidine-C_4_, just lacking the central C-C bond. The compound was soaked into ligand-free crystals of the *G. violaceous* P1 stem–loop (Supplemental Table S2), and the resulting crystals diffracted to 1.54 Å (Supplemental Table S3). The structure (PDB ID 6HBX) is shown in Figure 3. The structure of the stem–loop is closely similar to that bound to guanidine and methylguanidine (Fig. 3B; Huang et al. 2017a). The guanidine moieties are hydrogen bonded in the normal manner to O6 and N7 on the Hoogsteen edge of G9, with two guanidine N atoms donating hydrogen bonds to nonbridging O atoms of successive phosphate groups (Fig. 3C). The ethyl chains emerge from the same side openings as observed with bound methyl- and aminoguanidine and agmatine (Huang et al. 2017a). The terminal C atoms of the side chain are separated by 3.3 Å in this structure (Fig. 3D). However, the electron density is weak for the terminal atoms and the barrier to rotation about the N-C bonds to bring them to within bonding distance (1.5 Å) should be small.

### A crystal structure of the guanidine-II riboswitch bound to diguanidine-C_4_

**FIGURE 4.**
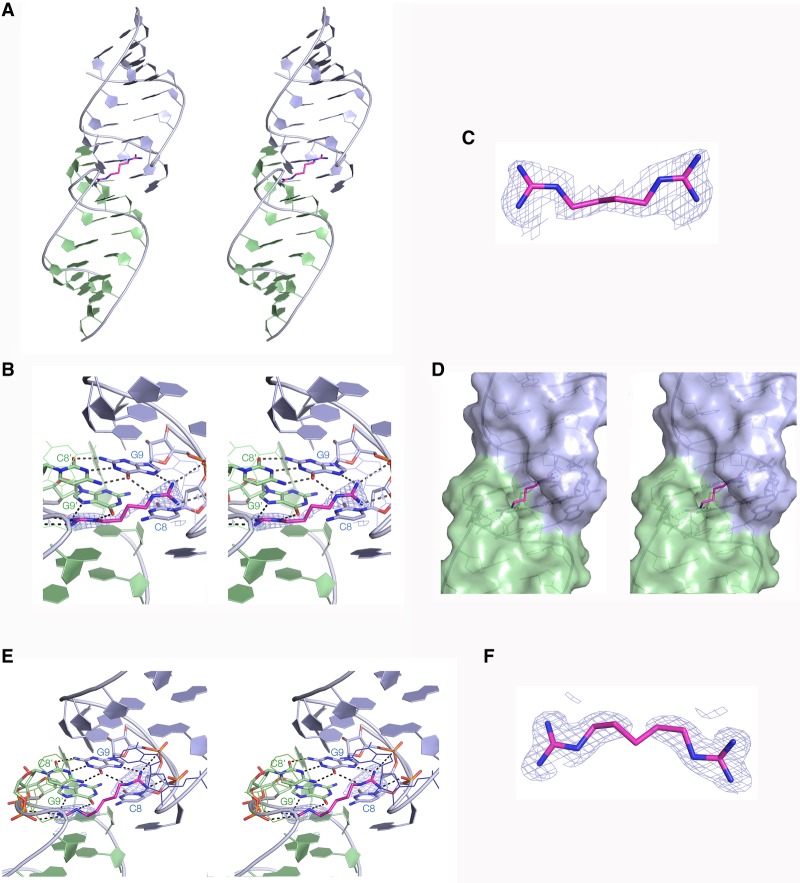
Crystal structures of diguanidine-C_4_ and diguanidine-C_5_ bound to *G. violaceous* riboswitch P1 stem–loop. (*A*) The overall structure of the P1 stem–loop dimer shown in parallel-eye stereoscopic view. The diguanidine-C_4_ ligand molecule is colored magenta. (*B*) Parallel-eye stereoscopic view of the diguanidine-C_4_ molecule bound at the dimer interface. Electron density (2*F*_o_−*F*_c_) contoured at 2σ is shown for the diguanidine-C_4_ molecule. Each guanidine moiety is hydrogen bonded to G9 and G9′ and nonbridging phosphate oxygens of the backbone. (*C*) The diguanidine-C_4_ with its experimental phasing electron density map contoured at 1σ. The position of the entire chain is defined, although the density for the central carbon atoms is weaker, indicative of some flexibility in this region. (*D*) A parallel-eye stereoscopic view of the dimer interface with a surface depicted for the riboswitch with bound diguanidine-C_4_. The polymethylene chain connecting the guanidine moieties is clearly visible emerging from the side openings of the binding pockets and traversing the minor groove side of the dimer interface. (*E*) A parallel-eye stereoscopic close-up view of the dimer interface with bound diguanidine-C_5_, with the electron density map (2*F*_o_−*F*_c_) for the ligand contoured at 2σ. Each guanidine moiety is hydrogen bonded to G9 and G9′ and nonbridging phosphate oxygens of the backbone. (*F*) The bound diguanidine-C_5_ molecule shown in isolation, with its experimental phasing electron density map contoured at 1σ. Electron density for the central carbon atom in the polymethylene linker is not visible, indicative of significant flexibility in the polymethylene chain.

Our chemically synthesized diguanidine-C_4_ was soaked into the *G. violaceous* P1 stem–loop crystals, and the resulting crystals diffracted to 1.66 Å (Supplemental Table S3). The structure was solved (PDB ID 6HBT; Fig. 4) and the position of the diguanidine-C_4_ ligand is well defined by the electron density map. This demonstrates that the two guanine moieties are bound in the usual manner (Fig. 4B), and are connected by the four-carbon methylene chain just as anticipated. The electron density for the C_4_ linker region is weaker compared to the guanidine groups (Fig. 4C). Moreover the crystallographic B-factors for these atoms are significantly higher (average value of 57.5) compared to those of the guanidino-C atoms (46.2). These data collectively suggest a greater flexibility of the C_4_ chain, most likely in a kind of crankshaft rotation. Figure 4D shows that the ligand emerges from the side opening of the binding pocket, traverses the minor groove and enters the binding pocket of the second site.

### A crystal structure of the guanidine-II riboswitch bound to diguanidine-C_5_

We also soaked our chemically synthesized diguanidine-C_5_ into the *G. violaceous* P1 stem–loop crystals, and the structure was solved to a resolution of 1.41 Å (PDB ID 6HC5; Fig. 4; Supplemental Table S3). The position of the two guanidine groups is well defined, bound in the normal manner (Fig. 4E). Electron density for the guanidines and the first two C atoms of the linker is clear, but that for the central C atom cannot be observed at a contour level of σ = 1.5 (Fig. 4F). The B-factor for the central C atom is 86.0, and the mean value for those flanking it is 50.2. The ligand is clearly bound in the expected manner, but the longer linker is more mobile. Our calorimetric measurements (Supplemental Table S1) show that lower free energy of binding of diguanidine-C_5_ compared to diguanidine-C_4_ is entirely due to a greater TΔS, consistent with a higher conformational entropy of the linker.

### Conclusion

The work described here clearly demonstrates that the two guanidine ligands of the guanidine-II riboswitch can be covalently linked by a chain of four or five methylene carbon atoms that pass through the side openings of the two ligand binding pockets located on the minor groove side of the dimeric riboswitch. The diguanidine ligands are hydrogen bonded into the two binding sites in the same manner as guanidine (Huang et al. 2017a), or ethylguanidine, although with subtle changes of position (Supplemental Fig. S3). The guanidine groups of diguanidine-C_5_ and ethylguanidine are within 0.2 Å, but those of diguanidine-C_4_ are retracted by 0.4 Å. This indicates that the shorter linker may be under some degree of tension.

We have measured the affinity of binding by calorimetry and fluorescence spectroscopy. Although there is some variation in the affinities measured using different methods, the diguanidine ligands bind with affinities that are consistently one order of magnitude higher than that of guanidine. Diguanidine-C_4_ has a slightly higher affinity than that of diguanidine-C_5_, most probably because of the greater flexibility of the linking chain in the latter. It is possible that rigidifying the linking chain could further increase binding affinity. The stoichiometry of the binding of the diguanidine ligands is consistent with binding as a single ligand. Thus, the results are entirely consistent with our structural understanding of the guanidine-II riboswitch and provide a new class of higher-affinity ligand.

The work here provides an example of structure-based ligand design using a natural riboswitch. The guanidine-II riboswitch ACGR stem–loop is one of the smallest riboswitches and so easy to combine with other RNA elements. The combination of this with diguanidine ligands has a number of potential applications in chemical and synthetic biology and RNA-based nanotechnology. For these, the diguanidine-C_4_ has a number of important properties. The compound binds to the riboswitch-derived RNA with higher affinity compared to guanidine, and is a nonnatural compound so chemically orthogonal to cellular metabolism. Importantly, diguanidine-C_4_ has low toxicity in rats (Ceretta et al. 2008), and is much less toxic than guanidine. This specific ligand-induced RNA interaction could be generally applicable in RNA technology, RNA design, and perhaps RNA-based therapeutics.

## MATERIALS AND METHODS

### Synthesis and characterization of diguanidine compounds

Diguanidine compounds were synthesized by guanylation of amines using 1-H-pyrazole-1-carboxamidine hydrochloride, following procedure B of Bernatowicz et al. (1992) (Supplemental Fig. S1). NMR spectra were recorded using a Bruker Avance DPX 400 spectrometer (^1^H at 400 MHz; 9.4 T) using automatic tuning and matching. Chemical shifts (δ) are expressed in ppm recorded using the residual solvent peak at 4.7 ppm as the internal reference in both cases. Signal splitting patterns are described as triplet (t), quintet, multiplet (m), or a combination thereof. Coupling constants (*J*) are quoted to the nearest 0.1 Hz. Fast atom bombardment (FAB) mass spectrometry was performed using an Agilent G6470A Triple Quadrupole spectrometer in positive mode of detection. A syringe pump was used to deliver methanol solutions of the diguanidine compounds (1.0 µg/mL) with a flow rate of 0.5 mL/min. A stainless-steel capillary was held at a potential of 3.0 kV. Nitrogen was used as nebulizer gas at a flow rate of 7.0 L/min, pressure 40 PSI at 350°C. Reported spectra are the averages of 15 scans using 500 msec accumulation time.

*N,N′-(butane-1,4-diyl)bis guanidinium (diguanidine-C_4_).* Agmatine sulfate (85 mg, 370 µmol), 1-H-pyrazole-1-carboxamidine hydrochloride (55 mg, 370 µmol), and 0.75 mL of 1.0 M Na_2_CO_3_ were stirred overnight at room temperature. The white precipitate was collected and washed three times with 1 mL MeOH/H_2_O (1:1) then dried in vacuo to yield 54 mg (53%). ^1^H NMR : 3.13 (4H, m, (CH_2_)_2_), 1.56 (4H, m, (CH_2_)_2_) (Supplemental Fig. S2A). FAB mass spectrometry gave a molecular mass of 173.10 (calculated 173.25) (Supplemental Fig. S1B).

*N,N′-(pentane-1,5-diyl)bis guanidinium (diguanidine-C_5_).* Cadaverine hydrochloride (100 mg, 590 µmol), 1-H-pyrazole-1-carboxamidine hydrochloride (170 mg, 1.18 mmol) and 2.4 mL of 1.0 M Na_2_CO_3_ were stirred overnight at room temperature. The white precipitate was collected and washed three times with 1 mL MeOH/H_2_O (1:l) then dried in vacuo to yield 110 mg (74%). ^1^H NMR : 3.10 [4H, t, J 6.3 Hz, (CH_2_)_2_], 1.53 [4H, quintet, J 6.9 Hz, (CH_2_)_2_], 1.36–1.28 (2H, m, CH_2_) (Supplemental Fig. S2B). FAB mass spectrometry gave a molecular mass of 187.10 (calculated 187.25) (Supplemental Fig. S1C).

### Synthesis of RNA oligonucleotides

RNA oligonucleotides were synthesized using solid-phase *t*-BDMS phosphoramidite chemistry (Beaucage and Caruthers 1981) as described in Wilson et al. (2001), implemented on an Applied Biosystems 394DNA/RNA synthesizer. Oligonucleotides containing 5-bromocytidine (ChemGenes) were deprotected in a 25% ethanol/ammonia solution for 36 h at 20°C. The oligonucleotide containing 2-aminopurine (Glen Research) was deprotected in 1:1 ammonia/methylamine solution for 20 min at room temperature followed by 10 min at 65°C. All oligoribonucleotides were redissolved in 100 μL of anhydrous DMSO and 125 μL triethylamine trihydrofluoride (Aldrich) to remove *t*-BDMS groups, and agitated at 65°C in the dark for 2.5 h. After cooling on ice for 10 min, the RNA was precipitated with 1 mL of butanol, washed twice with 70% ethanol and suspended in double-distilled water.

RNA was purified by gel electrophoresis in polyacrylamide under denaturing conditions in the presence of 7 M urea. The full-length RNA product was visualized by UV shadowing. The band was excised and electroeluted using an Elutrap Electroelution System (GE Healthcare) into 45 mM Tris-borate (pH 8.5), 5 mM EDTA buffer for 8 h at 200 V at 4°C. The RNA was precipitated with ethanol, washed once with 70% ethanol, and suspended in double-distilled water.

### Isothermal titration calorimetry

ITC titrations were performed at 298 K using an ITC-200 microcalorimeter (GE Healthcare). RNA solutions (30–60 µM) were prepared by diluting concentrated stocks into the binding buffer containing 40 mM HEPES (pH 7.2), 100 mM KCl, 10 mM MgCl_2_. Guanidine and diguanidine compounds were prepared in the same binding buffer with a concentration of 0.5–1 mM. Solutions were degassed for 2–5 min before loading. The sample cell was filled with 200 µL of RNA. Guanidine or diguanidine was injected in a volume of 0.4 µL for the first injection and 2 μL for the next 19 injections using a computer-controlled 40 µL microsyringe with an injection interval of 120 sec. Titration of ligands into the binding buffer or titration of the binding buffer into the RNA solution resulted in negligible evolution of heat. Integrated heat data were analyzed using a one-set-of-sites model in MicroCal Origin following the manufacturer's instructions. The first data point was excluded in analysis. The binding parameters Δ*H* (reaction enthalpy change in cal mol^−1^), *K* (binding constant in M^−1^), and *n* (bound ligands per RNA) were variables in the fit. The binding free energy Δ*G* and reaction entropy Δ*S* were calculated using the relationships Δ*G* = −*RT* ln *K*, where *R* = 1.987 cal mol^−1^ K^−1^, *T* = 298 K and Δ*G* = Δ*H* − *TΔS*. The dissociation constant *K*_d_ was calculated as 1/*K*.

### Fluorescence spectroscopy

Fluorescence spectra were recorded in 10 mM Tris-HCl (pH 8.0), 50 mM NaCl, and 10 mM MgCl_2_ at 25°C using an SLM-Aminco 8100 fluorimeter. The spectra were corrected for lamp fluctuations and instrumental variations, and polarization artifacts were avoided by crossing excitation and emission polarizers at 54.7°. Steady-state fluorescence emission spectra were recorded between 330 nm and 460 nm in 1 nm intervals with excitation at 315 nm. Spectra were integrated between 355 and 375 nm.

### X-ray crystallography

The *G. violaceus* P1 stem–loop RNA sequence used for crystallization was (5′–3′) GGUGGGGACGACCCCA(BrC)C where BrC is 5-bromocytosine. A solution of 1 mM RNA in 5 mM HEPES (pH 7.6), 100 mM KCl was heated to 95°C for 1 min. The solution was slowly cooled to 20°C and MgCl_2_ added to a final concentration of 2 mM. Ligands were soaked into crystals of the ligand-free P1 RNA using the conditions indicated in Supplemental Table S2. All the crystals were cryoprotected using mother liquid with an additional 25%–30% glycerol.

Diffraction data were collected on beamlines I04 and I03 of Diamond Light Source (Harwell, UK). Data were processed by XIA2 (Winter et al. 2018). The resolution cutoff for the data was determined by examining by CC1/2 and density map as described previously (Karplus and Diederichs 2012). Initial phase information were acquired from the SAD data by locating the bromine atoms with Autosol in the PHENIX suite. Models were adjusted manually using Coot (Emsley et al. 2010) and subjected to several rounds of adjustment and optimization using Coot, phenix.refine, and PDB_REDO (Joosten et al. 2014). Model geometry and the fit to the electron density maps were monitored with MOLPROBITY (Chen et al. 2010) and the validation tools in Coot. The unbiased electron density maps were generated through Br-SAD phasing and density modification by Phenix AutoSol. Details of data collection and refinement statistics for the crystallographic data are shown in Supplemental Table S3.

## SUPPLEMENTAL MATERIAL

Supplemental material is available for this article.

## Supplementary Material

Supplemental Material
