# Supplementary material for: Structure-guided design of a high-affinity ligand for a riboswitch
Source: RNA. 2019 Apr;25(4):423–30. doi: 10.1261/rna.069567.118 (PMC6426286; doi:10.1261/rna.069567.118)
Supplement: Supplemental Material [file supp_25_4_423__index.html]

Structure-guided design of a high affinity ligand for a riboswitch — Structure-guided design of a high-affinity ligand for a riboswitch — Supplemental Material 

# Structure-guided design of a high-affinity ligand for a riboswitch

## Supplemental Material

- Supplemental\_Information.pdf
