# Supplementary material for: Structure-guided design of a high-affinity ligand for a riboswitch
Source: RNA. 2019 Apr;25(4):423–30. doi: 10.1261/rna.069567.118 (PMC6426286; doi:10.1261/rna.069567.118)
Supplement: Supplemental Material [file supp_069567.118_Supplemental_Information.pdf]

Structure-guided design of a high affinity ligand for a riboswitch

L. Huang, J. Wang, T. J. Wilson and D. M. J. Lilley

SUPPLEMENTARY MATERIALS

SUPPLEMENTARY FIGURES

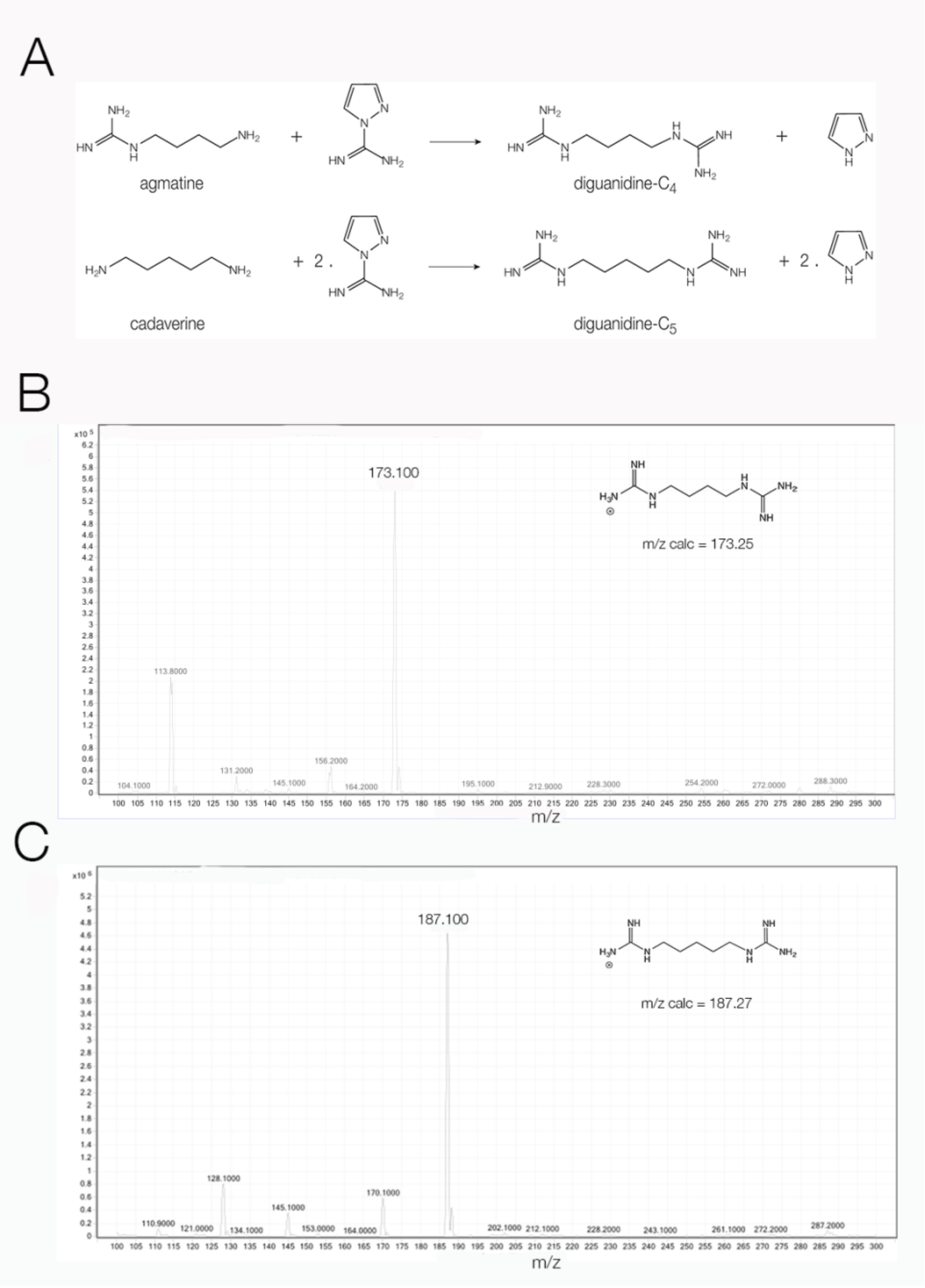

Figure S1. Synthesis and characterization of diguandine-C_4 and diguandine-C_5.

A. Schemes showing the synthetic reactions used to make the diguanidine compounds. Diguanidine-C_4 and diguanidine-C_5 were synthesized by guanylation of (4-aminobutyl)guanidine, (agmatine) and 1,5-diaminopentane (cadaverine) using 1 and 2 molar equivalents of 1-H-pyrazole-1-carboxamidine hydrochloride.

B and **C**. Characterization of diguandine-C_4 and diguandine-C_5 by mass spectrometry. Fast atom bombardment mass spectrometry using an Agilent G6470A Triple Quadrupole spectrometer in positive mode of detection of **A**. diguandine-C_4 and **B**. diguandine-C_5. The major species have molecular masses of 173.10 and 187.10 respectively, corresponding to the loss of a proton from each dianionic species, shown as inserts.

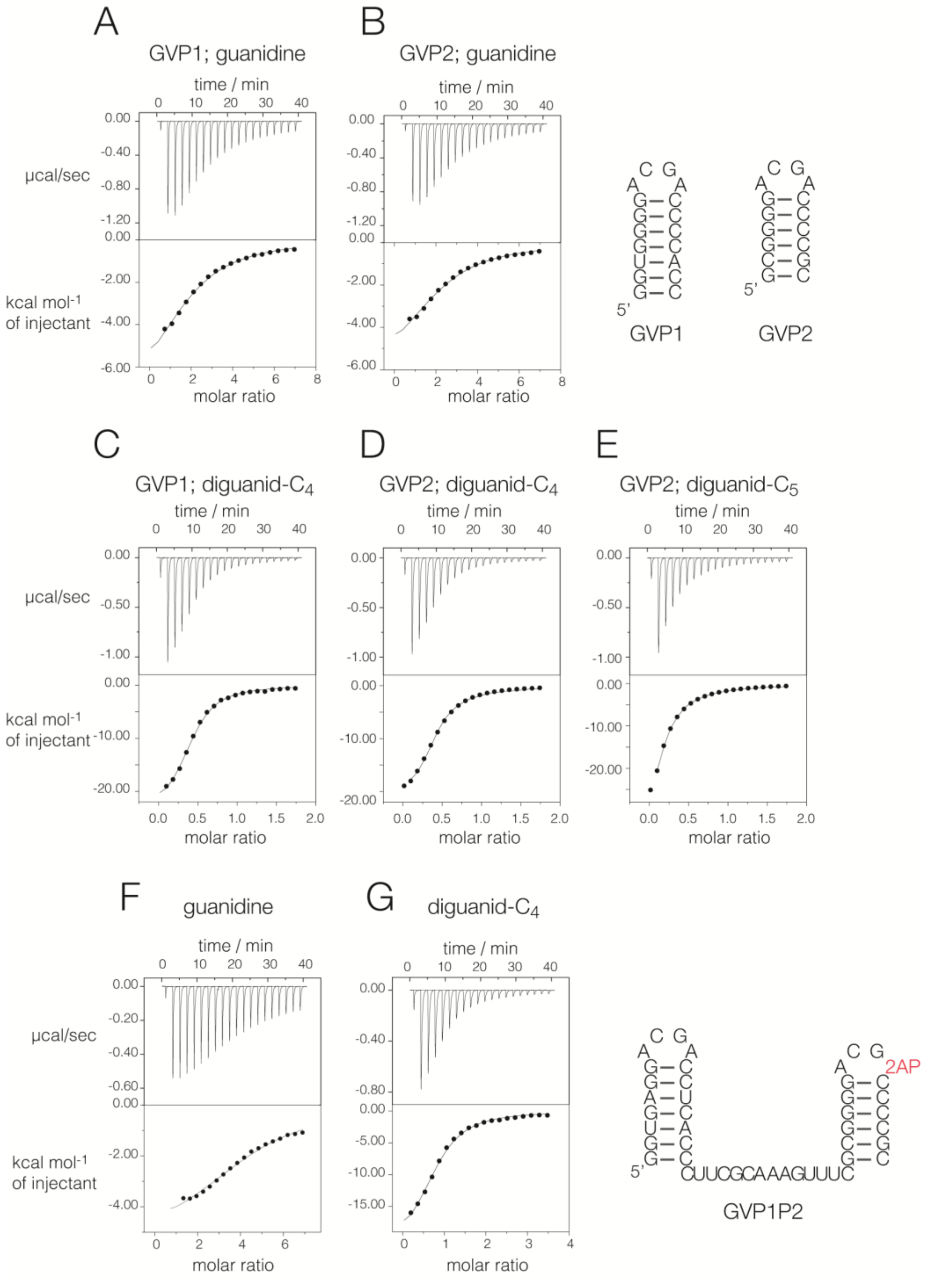

Figure S2. Binding of guanidine and diguanidine species to the *G. violaceus* guanidine-II riboswitch studied by isothermal titration calorimetry.

A and **B**; guanidine binding to the individual stem-loops GVP1 and GVP2 respectively (shown on the right).

C and **D**; diguanidine-C_4 binding to GVP1 and GVP2 respectively.

E; diguanidine-C_5 binding to the single stem-loop GVP1.

F and **G**; ITC analysis of ligand binding to the complete *G. violaceus* riboswitch with A10 2AP substitution in P2 (shown right, with 2AP highlighted in red). The RNA was titrated with **F**. guanidine and **G**. diguanidine-C_4 . The resulting thermodynamic data are given in Table S1.

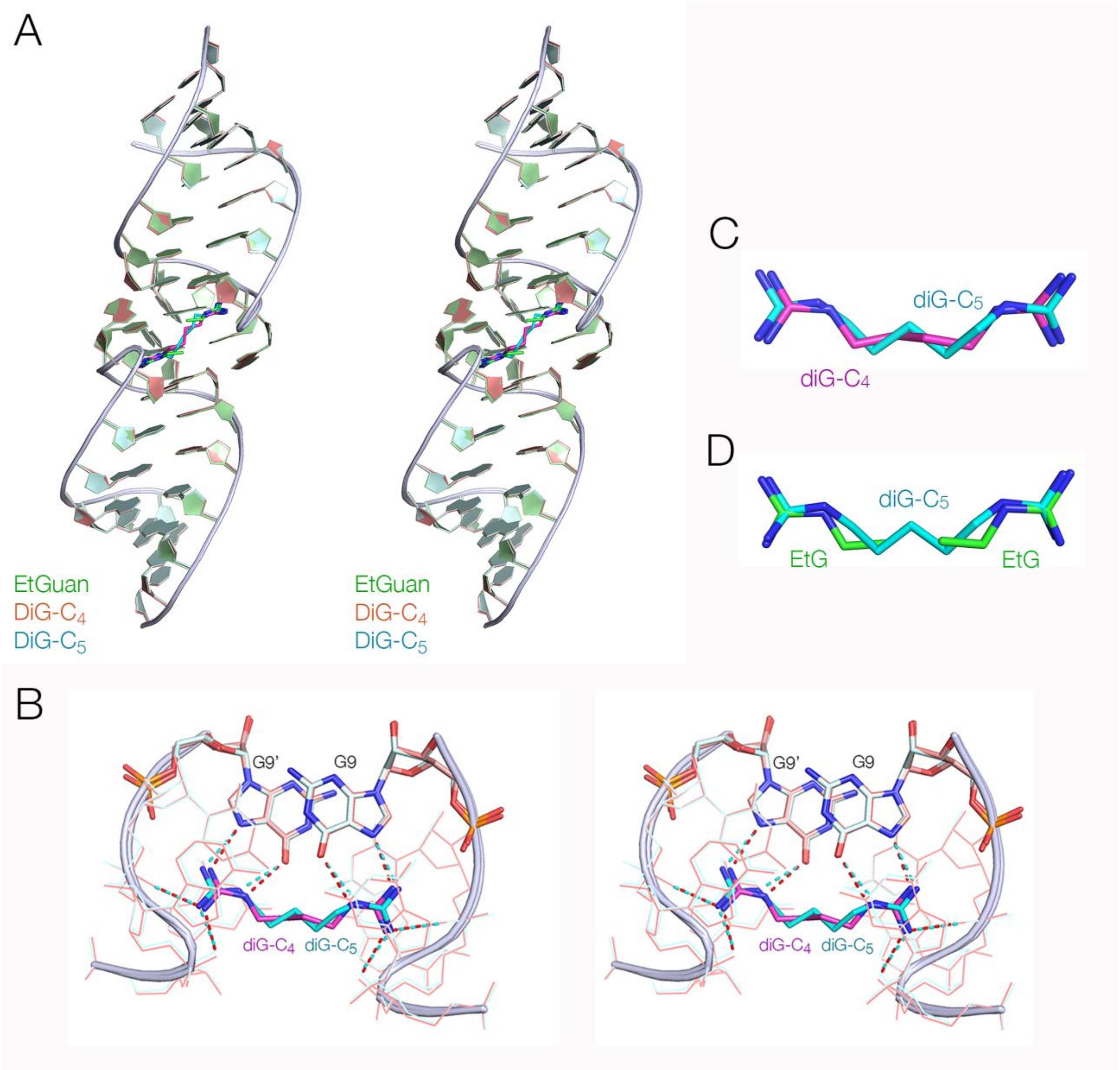

Figure S3. Superposition of *G. violaceous* riboswitch P1 stem-loop structures with ethylguanidine, diguanidine-C_4 and diguanidine-C_5 bound. The structures were aligned using the RNA alone.

A. An overall view of the three structures, colored ethylguanidine (green), diguanidine-C_4 (salmon, with magenta ligand) and diguanidine-C_5 (cyan). A parallel-eye stereoscopic view is shown. The RNA structures superimposed with RMSD values ethylguanidine - diguanidine-C_4 = 0.276 Å and ethylguanidine - diguanidine-C_5 = 0.319 Å.

B. A parallel-eye stereoscopic close view of the binding sites for the diguanidine-C_4 and diguanidine-C_5-bound structures. Hydrogen bonding to RNA ligands is shown by the broken lines. Diguanidine-C_4 and its hydrogen bonds are shown in magenta, and diguanidine-C_5 and its hydrogen bonds are shown in cyan.

C. The relative positions of diguanidine-C_4 (magenta) and diguanidine-C_5 (cyan) in the superimposed structures. The separation between the guanidine carbon atoms of diguanidine-C_4 and diguanidine-C_5 are 8.1 and 8.9 Å respectively.

D. The relative positions of ethylguanidine (green) and diguanidine-C_5 (cyan) in the superimposed structures.

SUPPLEMENTARY TABLES

RNA	ligand	n	ΔH / kcal.mol^{-1}	ΔS / cal K^{-1}mol^{-1}	ΔG / kcal.mol^{-1}	K_d/μM
GVP1	guanidine	1.93±0.11	-8.14±0.6	-8.2	-5.7	68 ± 6
GVP1	diguanidine-C_4	0.41±0.01	-24.3±0.5	-57	-7.3	4.7 ± 0.2
GVP2	guanidine	2.18±0.13	-6.5±0.5	-2.8	-5.7	66 ± 8
GVP2	diguanidine-C_4	0.40±0.01	-23.8±0.6	-55.7	-7.1	5.9 ± 4
GVP1P2	guanidine	2.73±0.11	-10.8±0.6	-15.8	-6.1	33 ± 4
GVP1P2	diguanidine-C_4	1.06±0.03	-13.1±0.6	-18.2	-7.7	2.2 ± 0.5
GVP1P2	diguanidine-C_5	1.22±0.04	-13.1±0.5	-19.8	-7.2	5.1 ± 0.8
GVP1P2 2AP	guanidine	4.32±0.13	-5.5±0.3	1.8	-6.0	41 ± 6
GVP1P2 2AP	diguanidine-C_4	0.79±0.02	-22.2±0.7	-50.7	-7.1	6.6 ± 0.6

Table S1. Thermodynamic parameters for ligand binding to guanidine II riboswitches and their individual stem-loops. The RNA names of the RNA species shown in the first column are derived from : GV = *G. violaceus*; P1 and P2 = individual stem-loops P1 and P2 respectively; P1P2 = P1 and P2 connected by 14 nt linker, i.e. complete riboswitch; 2AP complete riboswitch with P1 A10 2AP substitution (see Figure S3). ITC data for diguanidine -C_5 binding to GVP2 could not be fitted, and are not included in the table.

PDB ID	RNA	treatment	solution	resolution / Å	space group	beamline
6HBX	*G. violaceus* P1_7bp	soak 10 h 10 mM 1-ethyl - guanidine	0.01 M Mg acetate 0.01 M MES (pH 5.6) 2.5 M NH_4 acetate	1.54	$H3_2$	Diamond I04
6HBT	*G. violaceus* P1_7bp	soak 10 h 10 mM di - guanidine-C_4	0.01 M Mg acetate 0.01 M MES (pH 5.6) 2.5 M NH_4 acetate	1.66	$H3_2$	Diamond I04
6HC5	*G. violaceus* P1_7bp	soak 3 h 10 mM di - guanidine-C_5	0.01 M Mg acetate 0.01 M MES (pH 5.0) 2.5 M NH_4 acetate	1.41	$H3_2$	Diamond I03

Table S2. Summary of the RNA species, ligands and crystallization conditions used in these experiments, and the crystals obtained.

type	*G. violaceus* P1_7bp	*G. violaceus* P1_7bp	*G. violaceus* P1_7bp
ligands	1-ethylguanidine	diguanidine-C_4	diguanidine-C_5
PDB	6HBX	6HBT	6HC5
**Data collection**			
Space group	$H3_2$	$H3_2$	$H3_2$
Cell dimensions			
a, *b*, *c* (Å)	56.1, 56.1, 134.8	55.5, 55.5, 135.0	55.8, 55.8, 133.3
α, β, γ (°)	90 90 120	90 90 120	90 90 120
	SAD-Br	SAD-Br	SAD-Br
	Peak	*Peak*	*Peak*
Wavelength	0.9197	0.9197	0.9196
Resolution (Å)	28.04- 1.54 (1.57 - 1.54)	27.73 - 1.66 (1.69 - 1.66)	27.91- 1.41 (1.44 - 1.41)
R_{merge}	0.053 (1.436)	0.056 (1.316)	0.047 (1.92)
$I / \sigma I$	17.3 (1.2)	19.6 (1.3)	18.1 (0.8)
CC (1/2)	1.00 (0.48)	1.00 (0.57)	1.00 (0.43)
Completeness (%)	97.7 (96.5)	98.9 (97.1)	98.0 (100.0)
Redundancy	4.9 (5.1)	4.8 (5.0)	6.3 (6.5)
**Refinement**			
Resolution (Å)	28.03 – 1.54 (1.60 - 1.54)	26.92 - 1.69 (1.75 - 1.69)	27.91 – 1.41 (.146 - 1.41)
No. reflections	12127 (1168)	9671 (942)	15328 (2733)
R_{work} / R_{free}	0.204 / 0.239	0.206 / 0.214	0.177 / 0.199
No. atoms			
Macromolecules	364	364	364
ligands	34	43	40
B-factors			
Macromolecules	32.11	37.89	36.64
ligands	36.06	55.84	57.26
Solvent	45.36	44.22	49.68
R.m.s. deviations			
Bond lengths (Å)	0.009	0.006	0.012
Bond angles (°)	1.33	1.16	1.801

*Values in parentheses are for highest-resolution shell.

Table S3. Details of data collection and refinement statistics for the crystallographic data as deposited with the PDB.
